# Accuracy of the pre-hospital triage tools (qSOFA, NEWS, and PRESEP) in predicting probable COVID-19 patients’ outcomes transferred by Emergency Medical Services

**DOI:** 10.22088/cjim.11.0.536

**Published:** 2020

**Authors:** Peyman Saberian, Nader Tavakoli, Parisa Hasani-Sharamin, Maryam Modabber, Mahnaz Jamshididana, Alireza Baratloo

**Affiliations:** 1 Pre-hospital and Hospital Emergency Research Center, Tehran University of Medical Sciences, Tehran, Iran; 2 Department of Anesthesiology, Imam Khomeini Hospital Complex, Tehran University of Medical Sciences, Tehran, Iran; 3 Trauma and Injury Research Center, Iran University of Medical Sciences, Tehran, Iran; 4 Tehran Emergency Medical Service Center, Tehran, Iran; 5 Department of Emergency Medicine, Sina Hospital, Tehran University of Medical Sciences, Tehran, Iran

**Keywords:** COVID-19, Emergency Medical Services, Scoring System, Triage

## Abstract

**Background:**

This study aimed to evaluate the efficiency of pre-hospital triage tools including the qSOFA, NEWS, and PRESEP in determining the prognosis of probable COVID-19 patients.

**Methods:**

In this diagnostic accuracy study, all probable COVID-19 patients older than 16-year-old who were transferred to the hospital by the Tehran Emergency Medical Services (EMS) during the first month of the pandemic, entered to the study. The scores of qSOFA, NEWS, and PRESEP were calculated using data gathered while providing pre-hospital care. The primary outcome was death; and the secondary outcomes were ICU admission, length of stay in the ICU, and length of hospital stay.

**Results:**

The data of 557 individuals with the mean age of 56.93±18.31 were analyzed of whom 67.5% were males. The area under the ROC curve (AUC) of qSOFA, NEWS, and PRESEP for ICU admission was 0.553, 0.557, and 0.551, respectively. The AUC of qSOFA, NEWS, and PRESEP for death was 0.596, 0.566, and 0.604, respectively. The best obtained cut-off point for qSOFA was a score >0 (the sensitivity and specificity were 25.0 and 85.68%, respectively), for NEWS was a score >2 (the sensitivity and specificity were 83.61 and 32.67%, respectively), and for PRESEP was a score >1 (the sensitivity and specificity were 54.10 and 55.56%, respectively).

**Conclusion:**

Based on the findings of the current study, it is likely that the available pre-hospital triage tools (qSOFA, NEWS, and PRESEP) do not have proper efficacy to predict death, ICU admission, and disease severity of COVID-19 patients.

In December 2019, dozens of people were hospitalized in Wuhan, China due to an odd pneumonia, which the causative agent was subsequently called SARS-CoV-2, and the resulting disease is now known as COVID-19 (1). Despite the health measures taken, many countries are in crisis because of this virus. Significant increase in demand for health care facilities following the outbreak of this disease on the one hand, as well as contamination of health system staff with this virus, on the other hand, has led to the shortage of medical resources in many countries (2, 3). Limitations in capacity and access to resources in health care systems are so serious that they can lead to worse clinical outcomes during the exacerbation of the outbreak. Therefore, more appropriate use of available resources and revision of existing protocols to provide medical care to these patients at different levels are needed (4-6). The emergency medical service (EMS) has played an undeniable role as the first line of management of COVID-19 patients, which has been in great demand by the society since the beginning of this pandemic (7).

When health resources are limited, the use of predictive models for estimating patient’s risk or poor outcome, inappropriate triage of patients, and their transfer to medical centers can reduce the crowds of emergency departments (EDs) and the appropriate and optimal use of limited available resources (8). Various models have been proposed for appropriate health responses and outcome prediction of COVID-19 patients, but most of these models are based on hospital and laboratory data and have no application for the pre-hospital setting (9). Previously, pre-hospital triage scoring systems such as quick Sequential Organ Failure Assessment (qSOFA), Modified Robson Screening Tool (MRST), Modified Early Warning Score (MEWS) and Pre-hospital Early Sepsis Detection (PRESEP) and etc. have been assessed to determine the severity, long-term and short-term prognosis, and predicting the mortality of septic patients. However, either their practicality in out-of-hospital settings is still highly controversial or has not been used in particular for COVID-19 patients (10-15). Among them, guidelines from the National Institute for Health and Care Excellence (NICE) have currently suggested using the tool of National Early Warning Score-2 (NEWS2) to assess the risk of probable COVID-19 patients who may require hospitalization (16).

Given the impact of this pandemic on the capacity of pre-hospital and hospital systems, as well as the challenge of identifying high-risk individuals for transport to the hospital, the use of support systems and resources that can be useful to decide whether to transfer or not and predicting the prognosis of patients is inevitable. It seems that so far no study has assessed the accuracy of various tools predicting the prognosis of COVID-19 patients, or at least the results are not available before the present study. Therefore, this study aimed to evaluate the efficiency of pre-hospital triage tools including qSOFA, NEWS, and PRESEP in determining the prognosis of probable COVID-19 patients.

## Methods

***Study design: ***We performed a diagnostic accuracy cross-sectional study from February 19 until March 20 of 2020 using the data registry of the Tehran EMS center. To comply with the principles of confidentiality, all information was used anonymously. The study proposal was approved by the Ethics Committee of Iran University of Medical Sciences with the assigned code of IR.IUMS.REC.1399.225.

***Study population: ***All probable COVID-19 patients (based on WHO definition (17)) older than 16-year-old who were transferred to the hospital by the EMS during the study period, and were registered in the Tehran EMS, entered the study. Exclusion criteria were death at the scene, missing data of pre-hospital records, no access to the patient’s or his/her relative’s call number, and disagreement of the patient to participate in the study. Assuming a sensitivity of at least 65% for each tool, a 15% mortality rate in patients diagnosed with COVID-19 by the EMS technicians, an error of 10% for estimating sensitivity, and a 5% type-1 error of, the minimum sample size required was 550. During the first month of the COVID-19 outbreak in Iran, about 2600 suspected COVID-19 cases were registered in the Tehran EMS registry system, and the patients with a probability of one to five were systematically randomly selected.

***Scales definition: ***The accuracy of 3 prognostic prediction models designed for pre-hospital systems was evaluated in this study. The scores of qSOFA, NEWS, and PRESEP were calculated using data gathered from the Tehran EMS while providing pre-hospital care.

- qSOFA uses three criteria, assigning one point for systolic blood pressure (SBP) ≤100mm Hg, respiratory rate (RR) ≥ 22 breaths per min, and altered mentation (Glasgow coma scale<15). QSOFA ≥ 2 predict the increased risk of long-term hospitalization, ICU admission, or associated mortality (17).
- NEWS uses temperature, heart rate (HR), respiratory rate (RR), level of consciousness according to the AVPU, SPO2, and supportive oxygen. Its score varies between 0-20 (18).
- PRESEP uses these criteria, assigning one point for temperature> 38 and <36 °C, spo2 <92%, RR> 22 breaths/min, HR> 90 times per min, BP <90 mm Hg, and GCS score <15. Its score varies from 0-6 (14).

***Data gathering: ***The information of the patient’s pre-hospital phase was registered in a pre-prepared checklist. The required data including demographic information, chief complaint, accompanying symptoms, medical history, the result of initial assessments done by the emergency medical technician (EMT), and the final result at the scene are routinely recorded in the information system of the center. The patients were followed-up by phone call. The time of telephone follow-up was a week in April. Confirmed COVID-19 cases were diagnosed by reverse transcription polymerase chain reaction (RT-PCR) and/or lung computed tomography (CT) scan.

***Outcomes: ***The primary outcome of this study was death from the onset of symptoms until 2 months later, which was inquired through telephone follow-up of patients. The secondary outcomes were ICU admission, length of stay in the ICU, and length of hospital stay. The duration of hospitalization for more than 20 days or ICU admission was defined as the severe disease (due to the limited capacity of the ICU beds, it was possible not to be admitted to the ICU).

***Statistical analysis: ***The data were described with the Frequency (percent), Mean (standard deviation), and Quartiles as appropriate. 

The mean difference of quantitative variables in the two groups was assessed with the Independent t-test. In this study, three outcomes (ICU admission, severe disease, and death) and the accuracy of the qSOFA, NEWS, and PRESEP tools for the prediction of this outcome were assessed with the Receiver Operating Characteristic (ROC curve) analysis and the area under the ROC curve (AUC). The 95% confidence interval for AUC was calculated according to the DeLong et al. and Binomial exact method. The best cut-off point of each tool was calculated with the Youden J index based on the best sensitivity and specificity. Also, we calculated the sensitivity, specificity, positive and negative predictive value with a 95% confidence interval.

## Results

***Basic information: ***In this study, the data of 557 probable COVID-19 patients were analyzed. Totally, 181 (32.5%) were females and the rest were males. The mean age of the study patients was 56.93 years (SD=18.31). The basic information of the studied patients is presented in Table 1. In terms of vital signs, the respiratory rate (RR) ranged from 10 to 28 breaths per minute, the systolic blood pressure (SBP) ranged from 80 to 200 mm Hg, the diastolic blood pressure (DBP) ranged from 50 to 110 mm Hg, the pulse rate (PR) ranged from 50 to 180 beat per minute, and the O2 saturation (SpO2) ranged from 52 to 99%. Also, the Glasgow coma scale (GCS) ranged from 8 to 15; The GCS of 539 patients (97.1%) was 15, and less than 15 for the rest. 

**Table 1:** Basic information of suspected/confirmed COVID-19 patients

**Variable**	**Mean±SD**
**Age**	56.93±SD=18.31
SBP (mmHg)	120.97±15.69
DBP (mmHg)	76.19±8.58
PR (beats/min)	91.0±15.72
RR (breaths/min)	17.31±2.42
SpO2 (%)	90.16±6.48
Temperature (°C)	38.07±0.85
GCS	14.89±0.69
**Gender, N (%)**	
Male	376 (67.5)
Female	181 (32.5%)
**History of** ** chronic diseases, N (%)**	
Diabetes	71 (12.7)
Coronary heart disease	82 (14.7)
Respiratory diseases	38 (6.8)
None	224 (40.2)

SBP: systolic blood pressure; DBP: diastolic blood pressure; PR: pulse rate; RR: respiratory rate; SpO2: O2 saturation; GCS: Glasgow coma scale

Among them, 379 (68.0%) cases were further confirmed (using RT-PCR and/or lung CT scan) and 106 (19.0%) cases were not (although still maybe a disease case that due to the limitations of the used test were not diagnosed accurately), and the final diagnosis in 72 (12.9%) cases was unclear. The comparison of the baseline characteristics of the study patients, based on their final diagnosis, are mentioned in table 2, in which, only the length of hospitalization found to be statistically different (p<0.001).

**Table 2:** The comparison of the baseline characteristics of the study patients, based on their final diagnosis

	**Total (%)**	**RT-PCR and or lung CT scan**			**P-value**
		**Positive**	**Negative**	**Un-Known**	
Sex (male), n (%)	181 (32.5)	260 (68.6)	69 (65.1)	47 (65.3)	0.722
Age, mean (SD)	56.93 (18.3)	58.05 (17.7)	54.67 (20.0)	54.36 (18.3)	0.108
Length of hospitalization (day), mean (SD)	5.14 (7.1)	6.21 (7.8)	2.53 (4.4)	3.28 (5.4)	<0.001
ICU admission (day), mean (SD)	8.59 (7.4)	8.84 (7.6)	6.20 (5.1)	6.0 (3.6)	0.618
Death, n (%)	77 (13.8)	62 (16.4)	10 (9.4)	5 (6.9)	0.037

***Results related to the tools:*** Table 3 presents the details of the scales calculation. According to the qSOFA scale, 3.9% of subjects for GCS, 6.5% for RR, and 9.2% for SBP were positive. Based on the proposed cut-off point for this scale (score >1), the qSOFA test was positive for 10 (1.8%) patients in total, 78 patients obtained a score of 1, and the rest obtained 0. Among the measures of the NEWS, 2.9% of the patients had the most abnormalities in RR (≤8 or ≥25); 54.9% had the most abnormalities in SpO2 (≤91%), 32.9% needed supportive oxygen, and 6.7% had temperature ≥39.1 ˚C, 2.2% had the most abnormalities in SBP (≤90 or ≥220), 1.9% had the most abnormalities in PR (≤40 or ≥131), and 4.1% were unconscious. Finally, the NEWS for 413 patients before calculation, of which, the score of 22.0% of patients (n=91) was more than 6, in other words, these people have more clinical risk. The clinical risk for 0.5, 34.4, and 22.0% of patients were low, medium, and high, respectively. The mean score of this scale was 4.45 with a standard deviation of 2.72. Out of the 6 criteria of the PRESEP scale, the lowest and highest abnormalities were in SBP <90 mm Hg and SpO2 <92%, respectively. 

Only 1 (0.2%) patient had SBP <90 mm Hg, while 291(52.5%) patients had SpO2 <92%. Based on the proposed cut-off point for this scale (score >3), the PRESEP test was positive for 7 patients (1.7%) in total. 32.8, 24.4, and 16.7% of patients had a score of 1 to 3, respectively. For the assessment of the scales accuracy, any patient who did not have even one required variable was excluded. Therefore, the qSOFA, NEWS and PRESEP were calculated for 552, 413 and 412 patients, respectively.

**Table 3:** The details of scales calculation

**qSOFA**			**NEWS**			**PRESEP**		
**Variable**	**Value**	**N (%)**	**Variable**	**Value**	**N (%)**	**Variable**	**Value**	**N (%)**
GCS	<15	16 (3.9)	RR /min	≤8 or ≥25	9 (1.6)	RR /min	>22	12 (2.2)
	=15	397(96.1)		21-24	33 (6.0)		≤22	543(97.5)
RR /min	≥22	27 (6.5)		9-11	2 (0.4)	SpO2 %	<92	291(52.5)
	<22	386(93.5)		12-20	510(92.1)		≥92	263(47.5)
SBP mm Hg	≤100	38 (9.2)	SpO2*	≤91	291(52.5)	T °C	>38.0 or <36.0	183(44.0)
	>100	377(90.8)		92-93	114(20.6)		36.0-38.0	223(56.0)
				94-95	61 (11.0)	SBP mmHg	<90	1 (0.2)
				≥96	88 (15.9)		≥90	553(99.8)
			Supportive oxygen	Yes	355(64.1)	HR /min	>90	233(42.0)
				No	199(35.9)		≤90	322(58.0)
			T °C	≤35.0	0 (0.0)	GCS	<15	16 (2.9)
				≥39.1	28 (6.7)		15	539(97.1)
				35.1-36.0 or 38.1-39.0	163(39.2)			
				36.1-38.0	225(54.1)			
			SBP mm Hg	≤90 or ≥220	7 (1.3)			
				91-100	43 (7.8)			
				101-110	180(32.5)			
				111-219	324(58.5)			
			PR /min	≤40 or ≥131	10 (1.8)			
				111-130	42 (7.6)			
				41-50 or 91-110	185(33.3)			
				51-90	319(57.4)			
			Consciousness	Non alert**	16 (2.9)			
				Alert	539(97.1)			

SBP: systolic blood pressure; DBP: diastolic blood pressure; PR: pulse rate; RR: respiratory rate; SpO2: O2 saturation; GCS: Glasgow coma scale; T: temperature * on room air or supportive ** New-onset confusion, verbal/pain responsive, or unresponsive

**Figure 1:**
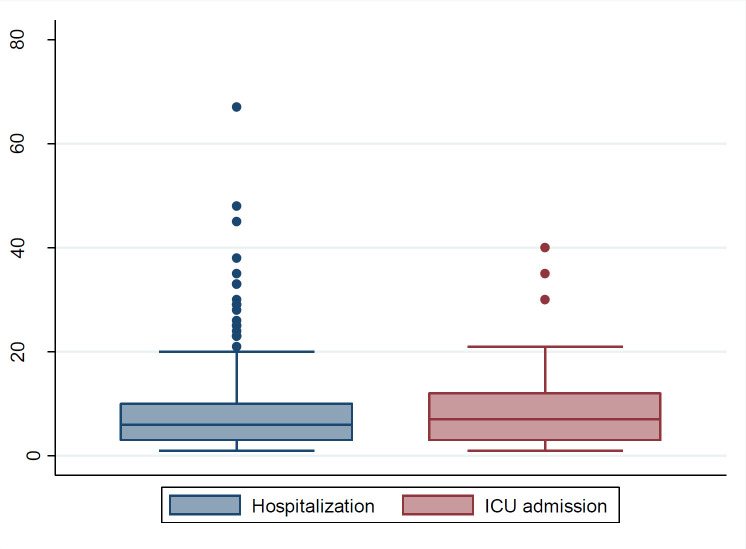
Distribution of length of hospitalization (day) and ICU admission (day)

***Results related to the final outcomes:*** Figure 1 shows the distribution of length of hospitalization (day) and ICU admission (day). Regarding the final diagnosis, as it was mentioned, the used tests (RT-PCR and/or lung CT scan) were positive in 379(68.0%) patients; they were negative in 106 (19.0%); and the final diagnosis was unclear in 72 (12.9%) cases. The duration of hospitalization ranged from 0 to 67 days with a mean value of 5.14 days (SD=7.14). Of all patients, 370 (66.7%) were hospitalized for at least one day, 85 (15.3%) were admitted to ICU. The length of ICU stay ranged from 1 to 40 days with a mean value of 8.59 days (SD=7.4). In this study, the duration of hospitalization over 20 days or ICU admission was considered as the severe illness, and accordingly, 90 (16.2%) patients had severe illness. Figure 1 shows the distribution of length of hospitalization histogram in the studied patients. Finally, a total of 77 (13.8%) patients died.

***Results of tools’ accuracy: ***The area under the ROC curve (AUC) of qSOFA, NEWS, and PRESEP for ICU admission was 0.553, 0.557, and 0.551, respectively. The AUC of qSOFA, NEWS, and PRESEP for death was 0.596, 0.566, and 0.604, respectively. Table 4 shows the AUC with a 95% confidence interval for three outcomes based on the three screening tools.

**Table 4:** The area under the ROC curve for predicting outcome divided in 3 scales in studied patients

**Outcome**	**Scale**	**AUC**	**Sig.** ^b^	**95% CI**	
				**Lower limit**	**Upper limit**
ICU admit	qSOFA	0.553	0.035	0.510	0.595
	NEWS	0.557	0.138	0.508	0.606
	PRESEP	0.551	0.188	0.502	0.600
Sever disease	qSOFA	0.558	0.017	0.516	0.600
	NEWS	0.574	0.045	0.525	0.622
	PRESEP	0.553	0.148	0.504	0.602
Death	qSOFA	0.596	<0.001	0.553	0.637
	NEWS	0.566	0.162	0.517	0.615
	PRESEP	0.604	0.009	0.555	0.652

b. Null hypothesis: true area = 0.5

The sensitivity and specificity of the qSOFA for predicting ICU admission based on the proposed cut-off point (score >1) were 2.38 and 98.27%, respectively. According to the Youden J index, the best obtained cut-off point for qSOFA was a score >0, for which the sensitivity and specificity were 25.0 and 85.68%, respectively, for NEWS, the best obtained cut-off point was a score >2, for which the sensitivity and specificity were 83.61 and 32.67%, respectively, and finally, for PRESEP, the best obtained cut-off point was a score >1, for which the sensitivity and specificity were 54.10 and 55.56%, respectively. The sensitivity and specificity of the qSOFA for predicting mortality based on the proposed cut-off point (score >1) were 6.67 and 97.95%, respectively. According to the Youden J index, the best obtained cut-off point was a score >0, for which the sensitivity and specificity were 32.0 and 86.58%, respectively. The sensitivity and specificity of the NEWS for predicting mortality based on the proposed cut-off point (score >6) was 33.96 and 79.72%, respectively and, according to the Youden J index, the best obtained cut-off point was a score >6. For PRESEP the best obtained cut-off point was a score >1, for which the sensitivity and specificity were 62.26 and 56.55%, respectively (Table 5).

**Table 5:** The statistical characteristics of scales at different cut-off points for predicting mortality and ICU admission in study patients

**Outcome**	**Scale**	**Cut-off**	**Sensitivity**	**Specificity**	**PPV**	**NPV**
			**(95% CI)**			
**Mortality**	qSOFA	>0*	32.00 (21.7-43.8)	86.58 (83.2-89.5)	27.3 (18.3-37.8)	89.0 (85.8-91.7)
	NEWS	>6*	33.96 (21.5-48.3)	79.72 (75.2-83.8)	19.8 (12.2-29.4)	89.1 (85.2-92.3)
	PRESEP	>1*	62.26 (47.9-75.2)	56.55 (51.2-61.7)	17.5 (12.3-23.6)	91.0 (86.5-94.4)
**ICU Admission**	qSOFA	>0*	25.0 (16.2-35.6)	85.68 (82.2-88.7)	23.9 (15.4-34.1)	86.4 (83.0-89.4)
	NEWS	>2*	83.61 (71.9-91.8)	32.67 (27.8-37.8)	17.7 (13.5-22.6)	92.0 (85.8-96.1)
	PRESEP	>1*	54.10 (40.8-66.9)	55.56 (50.2-60.8)	17.5 (12.3-23.6)	87.4 (82.4-91.5)

*Best cut-off point; CI: Confidence interval, PPV: Positive predictive value, NPV: Negative predictive value

## Discussion

Based on the presented results, none of the scales has acceptable diagnostic power to predict ICU admission and mortality of the disease. The predictive power of the PRESEP for mortality was >0.6 (medium predictive power). Regarding the current pandemic situation, increasing demand, and overcrowding of hospitals’ EDs, early identification of patients at risk of severe illness and decision-making about the level of care, especially in pre-hospital settings are very important.

Carr et al. performed a study in the United Kingdom that evaluated the NEWS2 for Covid-19, which showed that it had a poor predictive power for severe cases of COVID-19 (AUC = 0.628) but with the addition other factors such as age, CRP, neutrophil count, estimated GFR and albumin, its predictive power increased (AUC = 0.753). Most of the modeling studies have been performed on homogeneous Chinese populations, but this study has been conducted among several organizations and diverse populations that can be generalized (20). A study by a Chinese group suggested an adapted version of NEWS2 by adding an item of age <65 years old (3 points), indicating that increased age is associated with a poor prognosis (21). Respiratory failure is a hallmark in COVID-19 patients, which is often without circulatory failure (22). Several case reports described the presence of hypoxemia without evident symptoms of respiratory distress, so-called “silent hypoxemia” (23). The advantage of NEWS2 compared to other studied tools is that both hypoxia and supportive oxygen are included as scoring parameters. Despite the lack of evidence, the UK Royal College of Physicians recommends the use of the NEWS2 in the management of COVID-19 but emphasizes the fact that any increase in oxygen requirements should trigger further evaluation (16). Elderly patients have less typical and pronounced symptoms than younger ones, so the clinical risk score in these patients should be used with caution. A recently published case series of 17 patients aged ≥80 years shows that the variability in NEWS2 scores rather than a single observation at admission could predict poor outcome (24). The Jouffroy et al. study examined the accuracy of pre-hospital triage tools in predicting ICU admission in patients with septic shock and showed that the qSOFA, MRST, MEWS, or PRESEP scores were low in predicting ICU admission. According to the AUC, all performed tools were poor and had an AUC <0.7 (10). The study of Bhatraju et al. in COVID-19 patients with ARDS indicated that the mean score of the qSOFA on ICU admission was 1 and that patients on mechanical ventilation did not have a different qSOFA than patients without mechanical ventilation (25). Ferreira et al. also stated that the qSOFA is not suitable for identifying COVID-19 patients with poor results (26).

A study by Myrstad et al. showed that the strength of the NEWS2 score at hospital admission was higher than other clinical risk scores which are widely used in the prediction of severe disease and hospital mortality due to COVID-19 NEWS2 ≥6 AUC (0.790 , 95% CI 0.643-0.937), with 76.9% (95% CI 46.2-94.7) The SIRS, CRB-65, and qSOFA at hospital admissions are less able to predict the severity of COVID-19 and the obtained AUC were 0.624 (0.446-0.810), 0.584 (0.410-0.759), and 0.633 (0.470-0.796), respectively. So these clinical risk scores should be used with caution and with increased awareness of other clinical signs, especially respiratory distress and hypoxia in evaluating COVID-19 patients (27).

A systematic review conducted by Wynants et al. showed that variables such as age, history of chronic diseases, low lymphocyte count, and high lactate dehydrogenase are independent high-risk factors for COVID-19. However, it is mentioned that existing studies suffer several weaknesses including methodological weaknesses, selection bias, and reliance on cross-sectional data without accounting for censoring (9). Among the factors discussed in this study, except for age and the history of chronic diseases, all other items could not be evaluated in pre-hospital settings.

***Strengths and limitations: ***Our study examined existing models for predicting the prognosis of suspected patients to COVID-19 in the pre-hospital system and randomly selected patients with different characteristics that were distributed in various hospitals in Tehran, which could be generalized. However, some limitations must be acknowledged. First, the retrospective data registered in the Tehran EMS center were used, and there were no criteria to verify the validity of the recorded data. Second, some variables were missed, which were inevitably excluded from the study. Third, we used a two-month interval from the onset of symptoms to make a balance between the medium-term prognosis and the applicable risk stratification in the normal period of deterioration, but due to the involvement of more factors influencing the result, it will be more difficult to generalize the outcomes. Fourth, during the epidemic period, some patients were not transferred to the hospital after calling the ems for various reasons, including the patient's non-cooperation or reduction of the load of hospital visits, and these might be mild or moderate cases of the disease and were not evaluated in this study. Fifth, the time of the study was in late February and early March that was at the outbreak peak in the country, and this issue certainly had a great impact on the capacity of ICUs, though there were patients who were candidates for ICU admission but due to lack of vacant beds, were admitted to a normal ward equipped with ICU facilities.

In conclusion based on the findings of our study, it is likely that the current pre-hospital triage tools (qSOFA, NEWS, and PRESEP) do not have a proper predictive power for mortality, ICU admission, and disease severity in probable COVID-19 patients. However, the PRESEP had better mortality prediction power than the other two assessed scales, but still, its AUC was <0.7. So, there is a need to develop another scoring system in this regard.
